# Lipid profile in the aqueous humor of diabetic macular edema patients

**DOI:** 10.3389/fmed.2025.1541360

**Published:** 2025-02-14

**Authors:** Mengru Su, Qinglu Song, Ruiwen Cheng, Ye Zhang, Xinghong Sun, Feng Jiang, Qinghuai Liu

**Affiliations:** ^1^Department of Ophthalmology, Nanjing Drum Tower Hospital Clinical College of Nanjing Medical University, Nanjing, Jiangsu, China; ^2^Department of Ophthalmology, Nanjing Drum Tower Hospital, The Affiliated Hospital of Nanjing University Medical School, Nanjing, Jiangsu, China; ^3^Department of Ophthalmology, The First Affiliated Hospital of Nanjing Medical University, Nanjing, Jiangsu, China

**Keywords:** lipidomic, diabetic macular edema, macular disease, diabetic cataract, aqueous humor

## Abstract

Diabetic macular edema (DME) has become a global public health focus due to its increasing prevalence and significant impact on central vision. The aim of this study is to analyze the lipid profile characteristics of aqueous humor in DME patients and to identify differential lipid compounds that may serve as potential biomarkers for the pathogenesis and therapeutic intervention. A non-targeted lipidomics approach based on liquid chromatography–tandem mass spectrometry (LC–MS/MS) was used to analyze the lipid profiles of aqueous humor from patients with diabetic macular edema (DME group, 11 cases), diabetic cataracts (DC group, 14 cases), and age-related cataract (ARC group, 15 cases). The validation of identified lipid compounds through Orthogonal Partial Least Squares Discriminant Analysis (OPLS-DA) was conducted to examine possible varied lipid markers, setting the parameters of VIP > 1 and *p* < 0.05. Additionally, correlation network analysis, community classification, and functional enrichment analysis were performed on the differential lipids. 90 lipid compounds were identified, encompassing a range of 13 lipid categories. There were significant differences in the lipid profiles of the aqueous humor in DME. The lipid profile characteristics of aqueous humor in patients with DME are described for the first time. Compared to the control group. Various lipid metabolic disorders, such as sphingolipids particularly ceramide, phospholipids, and triglycerides, are involved in the pathogenesis of DME, and can be further studied as potential diagnostic and therapeutic lipid biomarkers.

## 1 Introduction

Diabetic retinopathy (DR), recognized as a significant microvascular issue in diabetes mellitus (DM), potentially resulting in vision loss and impacting independent living capabilities, has become a public health issue that is increasingly emphasized globally. Diabetic macular edema (DME) can occur at any stage of DR, severely impairing central vision and being a leading cause of vision impairment in the working-age population. The high prevalence of diabetes has caused a continuous increase in the number of DME patients, with the prevalence rising annually, making DME an urgent global public health issue that needs to be addressed (1). However, the pathogenesis of DME, which is complex, still remains incompletely understood. It is currently believed that DME results from an imbalance in fluid inflow and outflow. Elevated glucose levels lead to a rise in vascular endothelial growth factor (VEGF), boosting capillary permeability, breaking cell-to-cell connections, and harming the blood-retinal barrier (BRB), thus augmenting fluid flow into the retina (2). Prolonged high-glucose stimulation leads to chronic low-grade inflammation, directly and indirectly worsening BRB breakdown by activating Müller cells, retinal pigment epithelium (RPE) cells, and various inflammatory factors. This further stimulates the excessive production of inflammatory factors, causing edema in Müller and RPE cells, impairing drainage functions, reducing retinal fluid outflow, leading to fluid accumulation in the macular area, and eventually resulting in severe central vision loss (3).

Lipids participate in the formation of cell membranes, regulating the activity of transmembrane proteins, acting as intracellular and extracellular second messengers in signal transduction, and serving as an energy storage reservoir for cellular metabolism (4). According to the Lipid MAPS Alliance, individual lipid molecules are divided into eight categories. They are fatty acyls, glycerolipids, glycerophospholipids, sphingolipids, prenol lipids, sterol lipids, saccharolipids, and polyketides (5). The field of lipidomics focuses on detecting and measuring alterations in the lipidome through high-resolution mass spectrometry, with the goal of revealing the links between lipid metabolism and the diverse manifestations and results of diseases. It helps in the in-depth study of the pathogenesis and the search for therapeutic intervention targets. As we all know, the lipid metabolism and imbalance are potential risk factors for the occurrence and progression of diabetic complications. Moreover, they have a significant connection to the advancement of DR (6). Earlier research has broadened our knowledge of DME through the detection of cytokines, proteomics, metabolomics, and genomics in aqueous humor (7, 8). Nonetheless, the lipidomic study of aqueous humor in DME is lacking, and the precise function of lipid metabolism in DME remains unclear.

Various liquid samples, including serum and plasma, urine, cerebrospinal fluid, saliva, aqueous humor, vitreous humor, tears, can be used for lipidomics analysis (9). In clinical practice, it is relatively challenging to collect retinal tissue due to surgical constraints. Although vitreous fluid is easier to collect, strict control of surgical indications is necessary. Meanwhile, the aqueous humor plays an important role in the intraocular steady-state environment and is easy to sample, making it significant for studying retinal diseases. Lipid compounds with significant differences have been found in the aqueous humor of patients with diabetic cataracts (10), open-angle glaucoma (11), high myopia (12) and so on. Therefore, this study focuses on assessing and comparing the aqueous humor lipid profiles of patients with DME, DC and age-related cataracts (ARC). From the perspective of lipid metabolism, the study aims to uncover changes in lipid composition to explore potential lipid biomarkers and targets involved in the pathogenesis of DME.

## 2 Materials and methods

### 2.1 Patients

The study adhered to the Helsinki Declaration and was approved by the Ethics Committee of Nanjing Drum Tower Hospital (Approval No. 2023-423-01). Every participant provided their signed informed consent to collect medical data, surgical procedures, and samples of aqueous humor.

The research encompassed 40 patients, each undergoing treatment at the Ophthalmology Department of Nanjing Drum Tower Hospital between January 2023 and November 2023. This included 11 patients with diabetic macular edema (DME group, central subretinal thickness ≥ 300um), 14 diabetic cataracts without DME (DC group), and 15 nondiabetic patients with age-related cataracts (ARC group). We collected approximately 150 μL of aqueous humor samples before intravitreal injection or cataract surgery using a 30G needle and a 1 mL syringe at the corneal limbus. All collected samples were immediately frozen and stored at −80°C until analysis was required.

Patients were excluded if they had suffered from (1) a history of various ocular conditions, including glaucoma, high myopia, eye trauma, ongoing eye infections, and other retinal disorders leading to macular edema (such as retinal vein occlusion); records of intraocular surgery, photocoagulation, or injections (3). Uncontrolled high blood pressure and/or critical heart-related illnesses; prolonged use of systemic corticosteroids; ongoing treatment for other serious systemic conditions.

Gather medical information from patients, encompassing their age, gender, diagnosis, diabetes duration, and additional health background. Undertake an extensive and thorough eye examination before surgery, including tests such as best corrected visual acuity (BCVA), slit-lamp examination, intraocular pressure (IOP), ophthalmoscopy, ocular ultrasonic, and optical coherence tomography (OCT). The serological tests prior to the surgical procedure include fasting blood glucose (FBG) and lipid tests (total cholesterol, triglycerides, high-density lipoprotein (HDL), and low-density lipoprotein (LDL)). Questionnaires and medical record reviews are used to collect demographic details, clinical information, and medical backgrounds of the participants in the study.

### 2.2 Lipidomic analysis

#### 2.2.1 Lipid extract

The process of extracting lipids proceeds as follows:

1. Mix 50 μL of the aqueous humor specimen with 750 μL of a hybrid solvent [chloroform (CHORN China): Patel (DIKMA China) at a ratio of 2:1 v/v], and maintain the mixture at −20°C in a 2 mL centrifuge tube. Then, apply it for 30 s. Begin by chilling the test tube on ice for 40 min, followed by adding 190 μL of water and mixing in the vortex for another 30 s. Then, extend the incubation on ice for 10 min.
2. Perform a 5-min centrifugation at 12,000 rpm under standard temperature settings. Subsequently, move 300 μL of the organic layer into a newly placed centrifuge tube. Subsequently, incorporate 500 μL of a hybrid solvent (chloroform: methanol in a 2:1 volume ratio) and vortex for 30 s.
3. Conduct the centrifugation cycle again at 12,000 rpm for 5 min at room temperature, ensuring even distribution of 400 μL of organic matter into the centrifuge tube. The specimen stays concentrated in vacuum until it reaches complete dryness. Subsequently, the specimen is immersed in 200 μL of isopropanol (Thermo Fisher Scientific, USA) and passed through a 0.22 μm filtration membrane, resulting in the LC–MS prepared sample.

Centrifuge again at 12,000 rpm at room temperature for 5 min to ensure even distribution of the 400 μL of organic material into the centrifuge tubes. The samples are kept concentrated in a vacuum environment until completely dry. After this step, immerse the samples in 200 μL of isopropanol (Thermo Fisher Scientific, USA). Finally, obtain samples ready for LC–MS through a 0.22 μm filtration membrane.

#### 2.2.2 LC–MS

1. Chromatographic conditions: The process of chromatographic separation utilized an ACQUITY UPLC^®^ BEH C18 (2.1 × 100 mm, 1.7 μm, Waters) column, kept at a constant temperature of 50°C. The autosampler maintained a temperature of 8°C. The analytes were gradually eluted using a mixture of acetonitrile and water in a 60:40 ratio, and isopropanol and acetonitrile in a 90:10 ratio, maintaining a flow rate of 0.25 mL/min. Following the equilibration phase, each sample was administered a 2 μL injection.
2. Mass spectrometry condition: The ESI-MSn experiment used spray voltages of 3.5 kV (positive modes) and 2.5 kV (negative modes), respectively. The sheath gas was set to 30 arbitrary units, and the auxiliary gas was set to 10 arbitrary units. The capillary temperature was 325°C. The Orbitrap analyzer performed a scan over the mass range of 150–2,000 m/z with a resolution of 35,000 in full-scan mode. In the data-dependent acquisition (DDA) MS/MS experiment, we used the HCD scan technique. The energy of normalized collision was 30 eV.

### 2.3 Data processing and statistical analysis

The LipidSearch (13) software (V4.2.28) was employed to individually label the raw mass spectrometry data (*.raw format) for lipids, undertaking tasks such as peak alignment and filtering. Sum peak normalization served as the method for adjusting quantitative values to facilitate comparisons among various data scales.

Ropls R package was used to conduct principal component analysis (PCA), partial least squares discriminant analysis (PLS-DA), and orthogonal partial least squares discriminant analysis (OPLS-DA) to reduce the complexity of the sample data. To illustrate the variance in lipid composition across the samples, score plots, loading plots, and S-plots were created. The permutation test method was applied to check for model overfitting. *p* values were determined through statistical analysis, while the OPLS-DA technique was utilized to assess the significance of variables in projection (VIP) values. Fold change (FC) was calculated to assess the magnitude of component differences, measuring the strength and explanatory power of lipid content in classifying samples, and aid in the selection of marker lipids. Statistical significance for lipid molecules was established when *p* < 0.05 and VIP > 1.

Analyze lipid carbon chain length and saturation using R lipidomoR. Utilize a correlation network of diverse lipids for analysis and implement the Leiden community classification algorithm to segregate similar differential lipids into separate communities. For these lipids, perform a functional enrichment analysis with the R LION software package.

## 3 Results

### 3.1 Baseline characteristics

Table 1 encapsulates the demographic details of the participants. There were no noticeable variations found between the three groups in age, gender, duration of high blood pressure (HBP), BCVA and non-contact tonometer (NCT). However, compared to the ARC group, the DC and DME groups showed higher levels of FBC.

**Table 1:** Demographic characteristics of three group patients.

Characteristics	ARC (*n* = 15)	DC (*n* = 14)	DME (*n* = 11)	*p*-value	*Post Hoc*
Gender				0.529	
Male	5	5	6		
Female	10	9	5		
Age	69.07 ± 4.284	66.5 ± 7.144	63.27 ± 5.934	0.059	
Duration of DM	0 ± 0	11.357 ± 7.271	15.273 ± 6.389	0.000***	ARC<DC*** ARC<DME***
Duration of HBP	5.333 ± 8.121	5.786 ± 9.091	4.545 ± 8.202	0.728	
BCVA_logMAR	0.84 ± 0.651	1.219 ± 0.729	0.791 ± 0.556	0.145	
NCT	14.853 ± 2.038	15.371 ± 2.399	15.818 ± 3.175	0.668	
FBG	5.175 ± 0.578	6.953 ± 1.424	6.429 ± 1.584	0.000***	ARC<DC*** ARC<DME**
TG	1.443 ± 0.747	1.621 ± 0.869	1.31 ± 0.754	0.632	
TC	4.791 ± 1.2	5.179 ± 1.359	4.334 ± 1.123	0.231	
HDL	1.267 ± 0.258	1.233 ± 0.158	1.277 ± 0.478	0.583	
LDL	2.902 ± 0.967	3.104 ± 1.129	2.525 ± 0.8	0.410	

DM, diabetes mellitus; BCVA, best corrected visual acuity; NCT, non-contact tonometer; FBG, fasting blood-glucose; TG, triglyceride; TC, total cholesterol; HDL, high-density lipoprotein cholesterol; LDL, low-density lipoprotein cholesterol. *p < 0.05, **p < 0.01, ***p < 0.001.

### 3.2 Quality assurance and lipid clustering analysis

**Figure 1:**
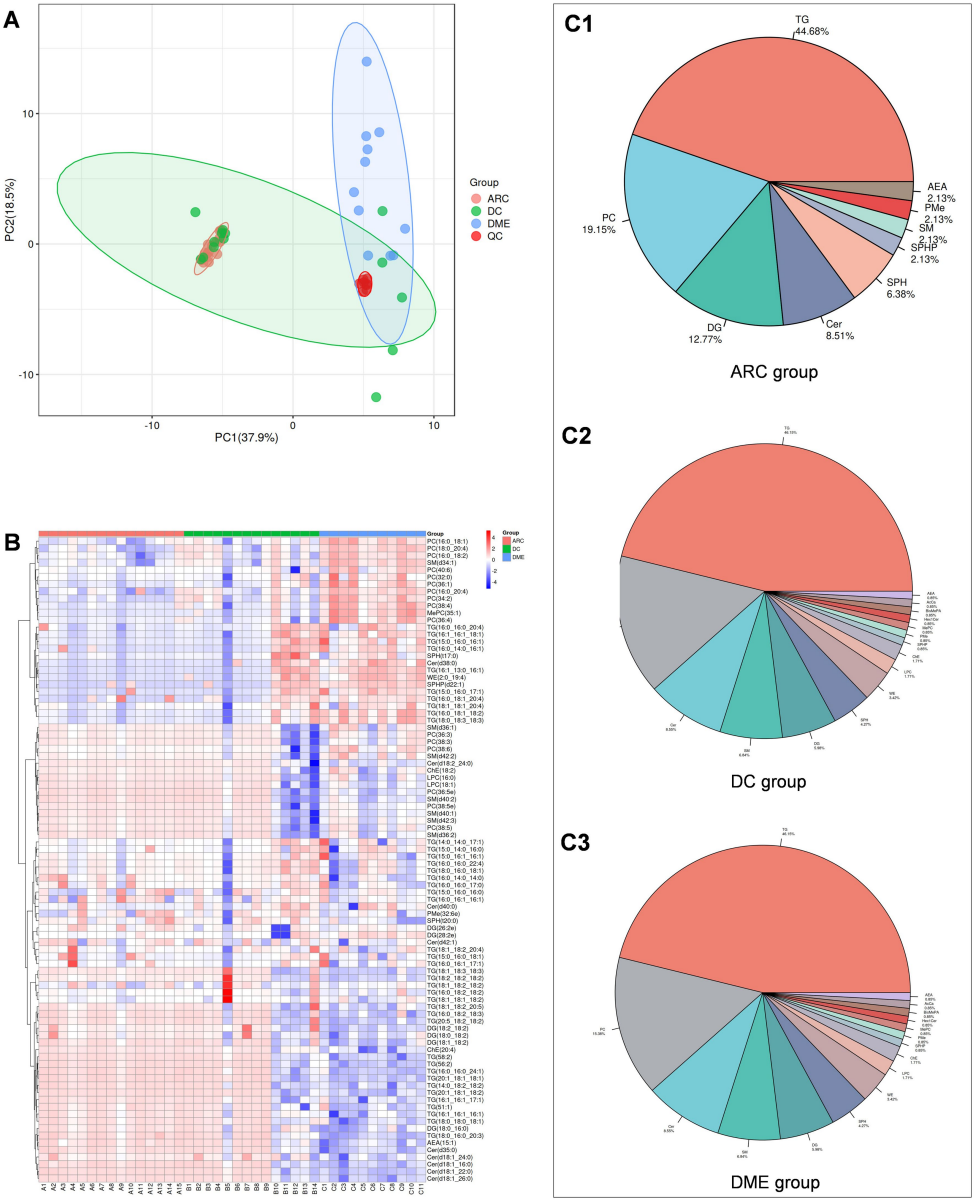
**(A)** QA-PCA score plot for three groups of samples. Red dots indicate quality control samples. **(B)** Overall lipid clustering heat map. The redder the color, the higher the expression level; the bluer the color, the lower the expression level. Columns represent samples, and rows represent lipids. The lipid clustering tree is on the left side. **(C1–C3)** are pie charts showing lipid classification.

First, we perform quality assurance -principal component analysis (QA-PCA) on the test results (Figure 1A), and then conduct a comprehensive clustering analysis of all detected lipids (Figure 1B) to gain an overview of the results. In total, 90 lipids were reliably detected across the three groups, involving 13 lipid classes. The distribution of various lipid classes is shown in Figure 1C. TG accounted for the highest percentage in the aqueous humor of all three groups. The percentage distribution of various lipids in DME and DC was consistent.

### 3.3 Lipid species analysis

**Figure 2:**
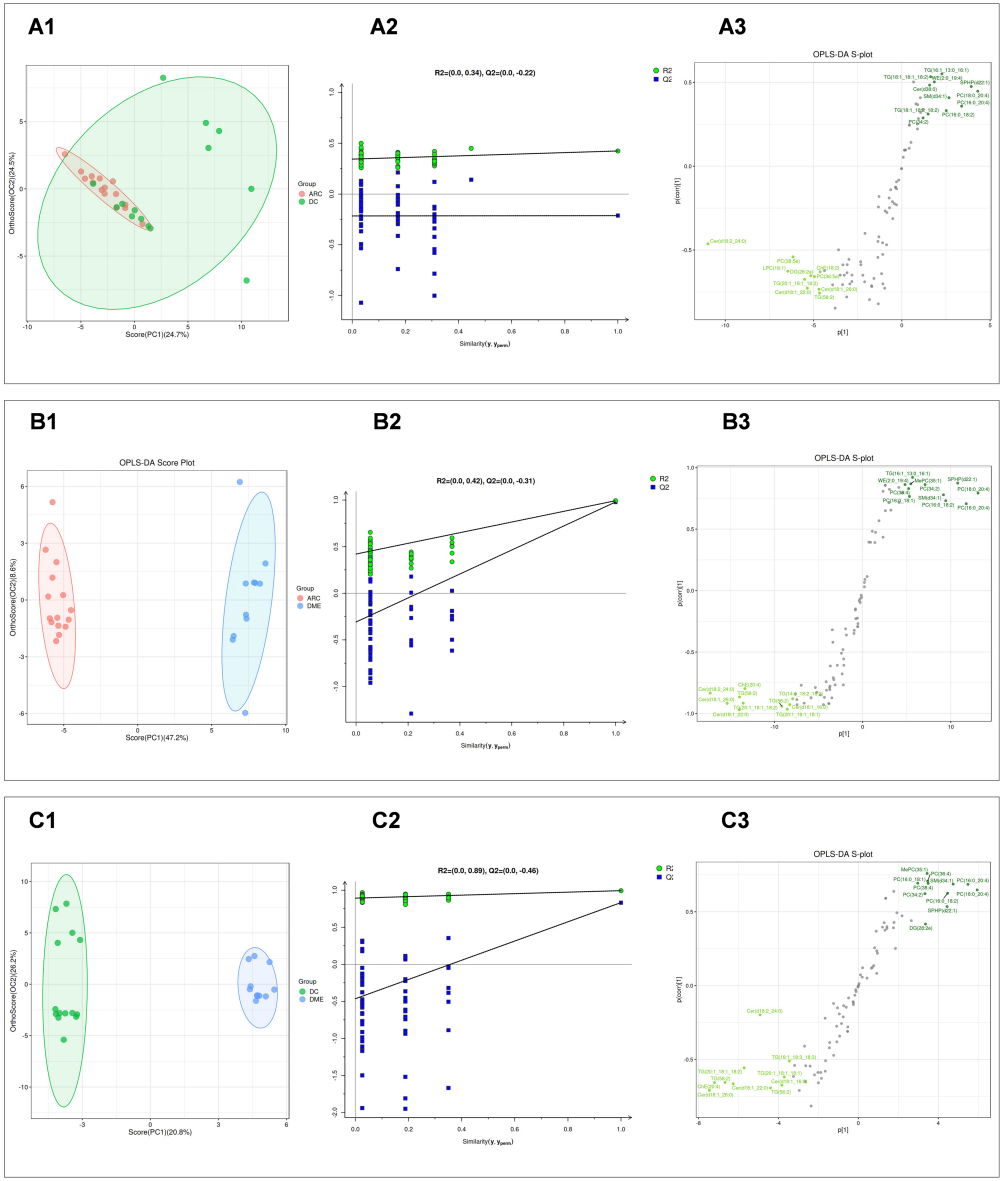
**(A1, B1, C1)** Display the OPLS-DA score plots for DC vs. ARC, DME vs. ARC, and DME vs. DC, respectively. The horizontal axis and the vertical axis represents the explained variance of the first principal component and the second principal component respectively. Each dot represents a sample. **(A2, B2, C2)** Present the OPLS-DA permutation test plots for DC vs. ARC, DME vs. ARC, and DME vs. DC, respectively. **(A3, B3, C3)** Illustrate the OPLS-DA S-plots for DC vs. ARC, DME vs. ARC, and DME vs. DC, respectively. The horizontal axis represents the correlation coefficient between the principal component and the lipid, while the vertical axis represents the correlation between the principal component and the lipid.

Subsequently, we performed the OPLS-DA for DME vs. ARC, DME vs. DC, and DC vs. ARC, obtained model validation parameters, and created OPLS-DA score plots graphs (Figures 2A1,B1,C1), permutation test plots graphs (Figures 2A2,B2,C2), and S-plots graphs (Figures 2A3,B3,C3) based on the analysis results. Our findings indicate a notable degree of fit and predictive ability for the model of DME vs. ARC, as well as for DME vs. DC, but the model validation parameters for the DC vs. ARC were inadequate (usually, R2 and Q2 values should surpass 0.5, with minimal differences between them. As the values of R2X and Q2 approach 1, the effectiveness of the model improves) (Table 2).

**Table 2:** The OPLS-DA model validation parameters.

Comparison	Pre	R2X (cum)	R2Y (cum)	Q2 (cum)
DC vs. ARC	1 + 1 + o	0.492	0.423	−0.213
DME vs. ARC	1 + 1 + o	0.558	0.992	0.978
DME vs. DC	1 + 5 + o	0.808	0.993	0.832

Pre: Number of principal components; R2X: Model’s explanatory power (Variant X dataset); R2Y: Model’s explanatory power (Variant Y dataset); Q2: Model’s predictability.

A comparative study was conducted, contrasting DC with ARC, DME with ARC, and DME with DC. The screening of differential lipid compounds was based on VIP >1 and *p* < 0.05 (Table 3). There were 52 lipid compounds in DME vs. ARC aqueous humor that showed significant differences and were of potential significance as biomarkers (Supplementary Table S1), and 39 lipid compounds in DME vs. DC aqueous humor that showed significant differences and were also of potential significance as biomarkers (Supplementary Table S2). Although the model validation for DC vs. ARC was not ideal, 27 differential lipid compounds were still screened (Supplementary Table S3).

**Table 3:** Statistical table of differential lipid compounds.

Comparison	Total	Up	Down	Total_DE
ARC vs. DC	90	2	25	27
ARC vs. DME	90	19	33	52
DC vs. DME	90	15	24	39

**Figure 3:**
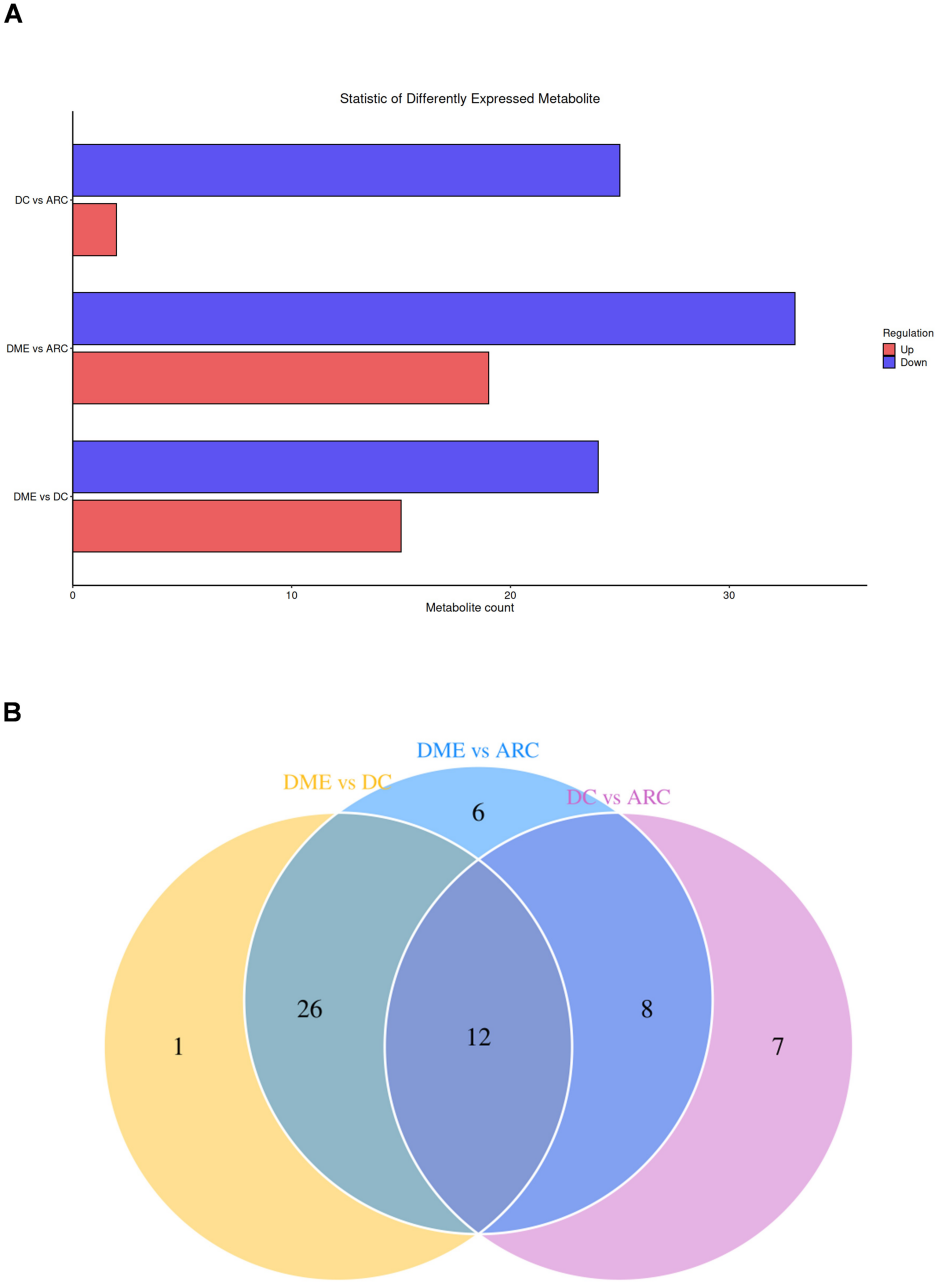
**(A)** Statistical bar chart of differential lipids between groups. The x-axis reveals the quantity of differential lipids, while the y-axis represents the different groups. **(B)** Venn diagram of differential lipids, with the overlapping region representing the common differential lipids.

The bar chart visually displays the changing trends of differential lipids between the two groups (Figure 3A). The DME vs. DC group and the DME vs. ARC group share 38 distinct lipid overlaps (Figure 3B). DG (18:0_18:2) shows significant downregulation only in the DME vs. DC group, with no significant difference observed in the DME vs. ARC group. Twelve differential lipids overlap among the DME vs. ARC, DC vs. ARC, and DME vs. DC comparisons, showing a consistent trend of change (Table 4).

**Table 4:** List of differential lipids overlapping in three groups.

Accession	Class	Regulation		
		DME vs. ARC	DME vs. DC	DC vs. ARC
Cer(d18:1_16:0)	Cer	Down	Down	Down
Cer(d18:1_22:0)	Cer	Down	Down	Down
Cer(d18:1_26:0)	Cer	Down	Down	Down
Cer(d35:0)	Cer	Down	Down	Down
TG(14:0_18:2_18:2)	TG	Down	Down	Down
TG(16:0_16:0_24:1)	TG	Down	Down	Down
TG(16:1_13:0_16:1)	TG	Up	Up	Up
TG(18:0_16:0_20:3)	TG	Down	Down	Down
TG(20:1_18:1_18:1)	TG	Down	Down	Down
TG(20:1_18:1_18:2)	TG	Down	Down	Down
TG(56:2)	TG	Down	Down	Down
TG(58:2)	TG	Down	Down	Down

**Figure 4:**
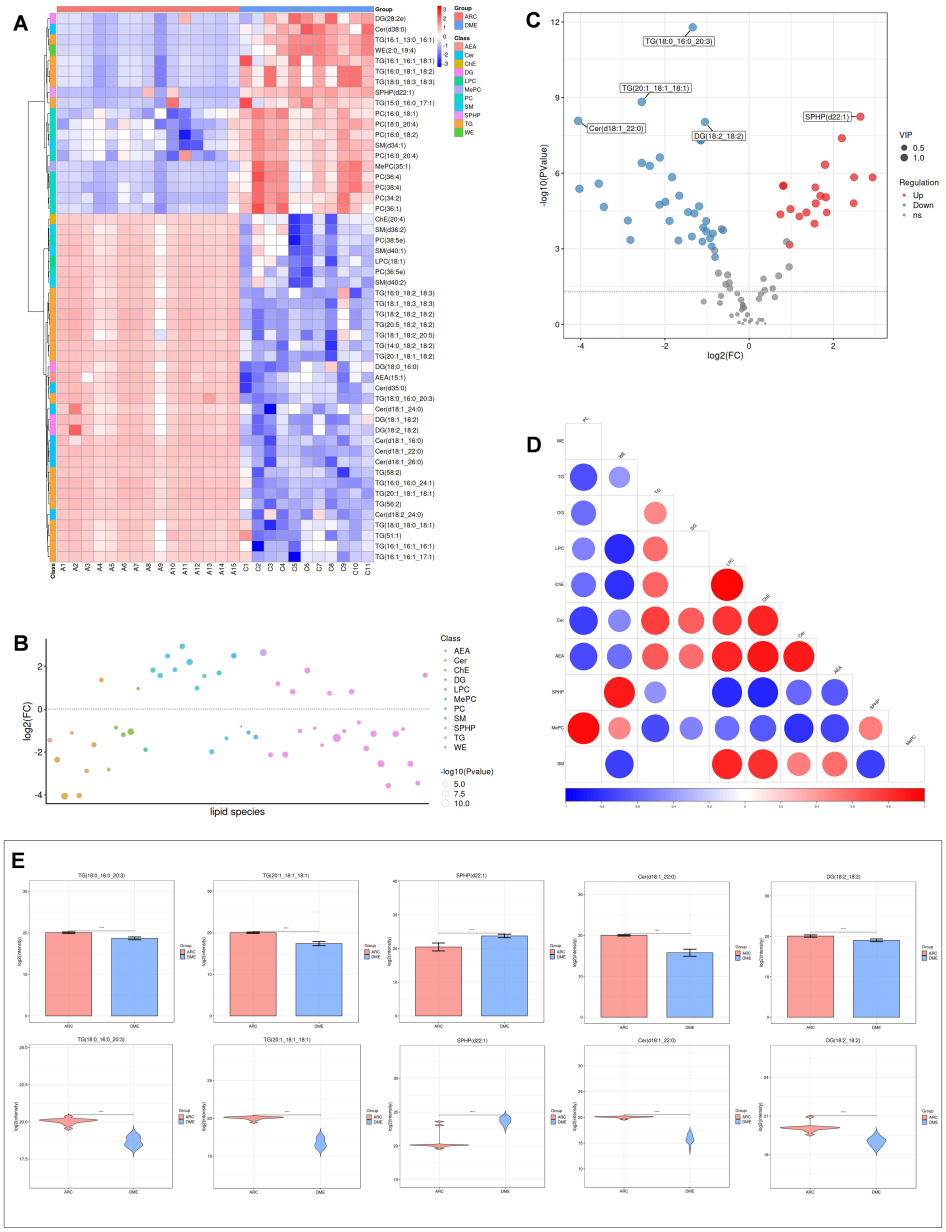
Differential lipid analysis of DME vs ARC. **(A)** Differential lipid class clustering heatmap. Red indicates higher expression levels, while blue indicates lower expression levels. Columns represent samples, and rows represent lipids. The lipid clustering tree is shown on the left. **(B)** Lipid classification bubble chart. Colors represent different lipid categories, and bubble size corresponds to the -log10 of the p-value. **(C)** Volcano plot of differential lipids. Each point represents a lipid, with red indicating upregulation, blue indicating downregulation, and gray representing lipids that did not pass differential screening. The top five lipids with the smallest p-values are labeled. **(D)** Heatmap of significantly correlated lipid classes. The horizontal axis represents the correlation coefficient, with larger dots indicating smaller p-values. Red indicates a positive correlation, blue indicates a negative correlation, and the color depth reflects the absolute value of the correlation coefficient. **(E)** Bar charts and violin plots of the five lipids with the smallest *p*-values. The horizontal axis represents the groups, while the vertical axis shows the range of lipid signal values. *p < 0.05, **p < 0.01, ***p < 0.001, ****p < 0.0001.

**Figure 5:**
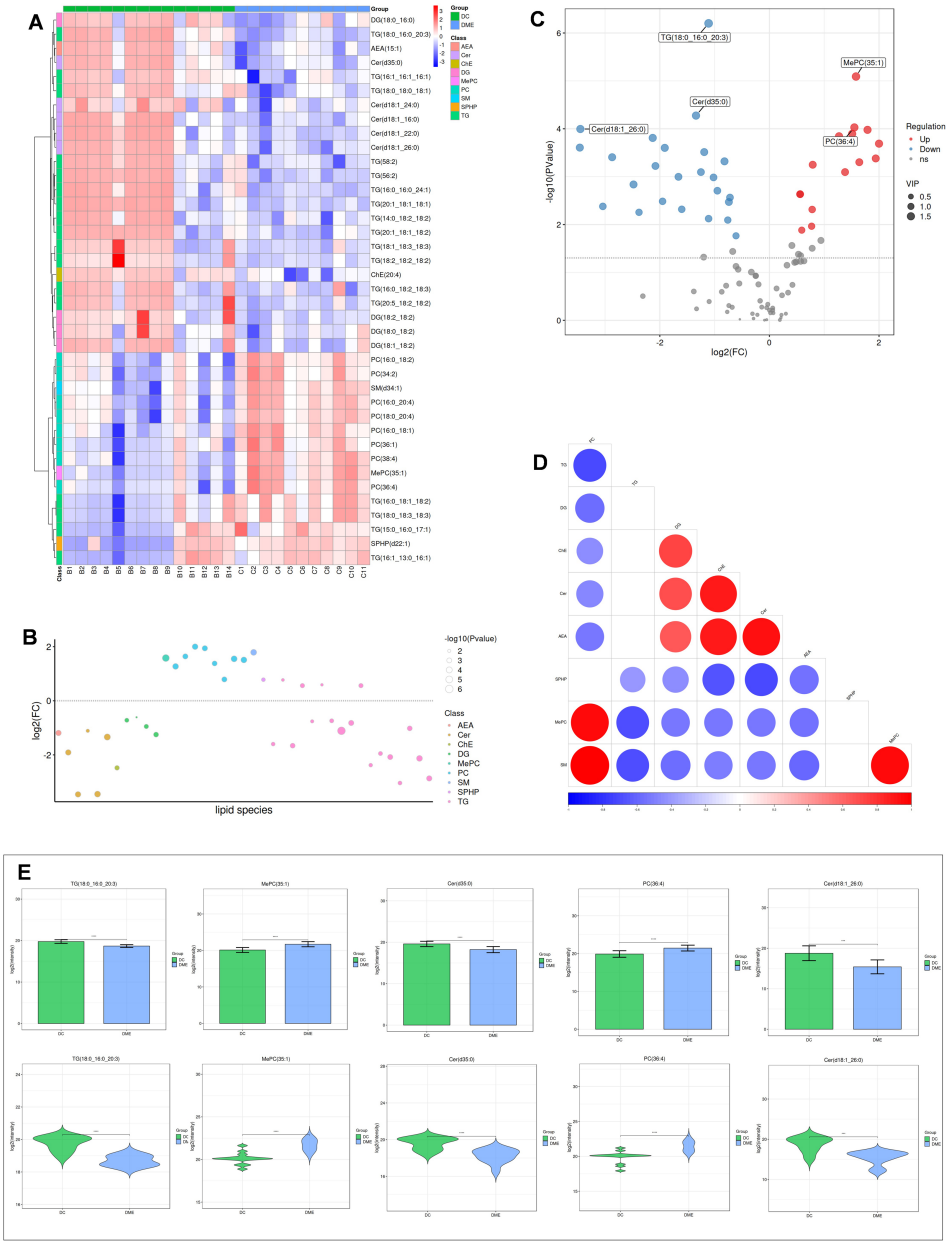
Differential lipid analysis of DME vs DC. **(A)** Differential lipid class clustering heatmap. **(B)** Lipid classification bubble chart. **(C)** Volcano plot of differential lipids. **(D)** Heatmap of significantly correlated lipid classes. **(E)** Bar charts and violin plots of the five lipids with the smallest *p*-values.

Heat maps were used to conduct and exhibit the cluster analysis comparing lipids in DME vs. ARC (Figure 4A) and DME vs. DC (Figure 5A). A bubble chart was created using the *p* values and FC values of the differential lipids to visually represent the concentration consistency and differences between various lipid classes. In the comparison of DME and ARC, AEA, Cer, DG, and LPC all show a downward trend, in contrast to ChE, MePC, PC, SPHP, and WE, which show an upward trend. Within the SM and TG classifications, there are variations in the expression patterns of different lipid configurations (Figure 4B). When comparing DME to DC, AEA, Cer, DG, and ChE all show a downward trend, whereas MePC, PC, SM, and SPHP show an upward trend. The expression trends of different TG configurations also vary (Figure 5B). The volcano plot shows the distribution of differential lipids in DME vs. ARC (Figure 4C) and DME vs. DC (Figure 5C), with the five most significant lipids highlighted. Next, we calculated the Pearson correlation coefficient between lipid classes and presented the synergistic interactions through a lipid class correlation heatmap. In the DME vs. ARC group, AEA, Cer, ChE, LPC, DG, and TG all show negative correlations with PC and MePC. MePC, in addition to being positively correlated with PC, also shows a positive correlation with WE and SPHP. AEA, Cer, ChE, LPC, and SM show a positive correlation among themselves, yet exhibit a negative correlation with WE and SPHP (Figure 4D). In the comparison of DME and DC, there is a positive correlation trend among SM, PC, and MePC, and they are negatively correlated with AEA, Cer, ChE, DG, and TG. AEA, Cer, ChE, and DG each exhibit positive interrelations and negative correlations with SPHP. SPHP is negatively correlated with TG (Figure 5D). Within the DME vs. ARC group, TG (18:0_16:0_20:3), TG (20:1_18:1_18:1), SPHP (d22:1), Cer (d18:1_22:0), and DG (18:2_18:2) emerge as the top five lipid compounds showing significant differences (Figure 4E). Meanwhile, in the DME vs. DC group, the five most distinct lipid compounds are TG (18:0_16:0_20:3), MePC (35:1), Cer (d35:0), PC (36:4), Cer (d18:1_26:0) (Figure 5E).

**Figure 6:**
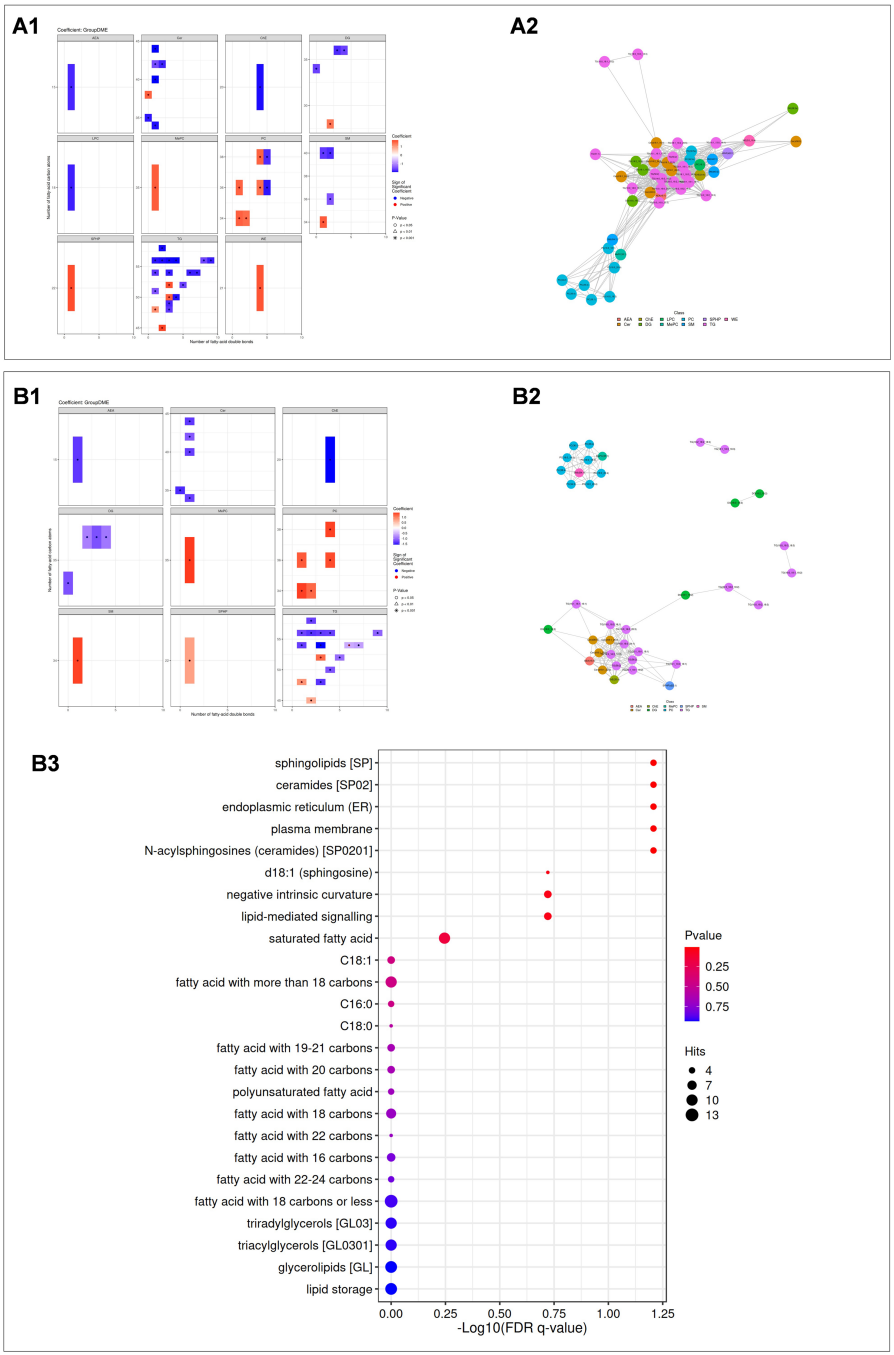
**(A1, B1)** Statistical heatmaps showing lipid structure differences between DME vs ARC **(A1)** and DME vs DC **(B1)**. Red indicates upregulation, blue indicates downregulation, and the depth of color reflects the significance of the differences. **(A2, B2)** Lipid correlation network diagrams for DME vs ARC **(A2)** and DME vs DC **(B2)**. Dots represent lipid molecules, with colors indicating their classifications. Lines connect lipids with significant correlations. **(A3, B3)** Enrichment analysis results for DME vs DC. A bubble chart is shown, with the horizontal axis representing the -log10 value of the enrichment FDR q-value, and the vertical axis displaying the names of structures and functions enriched in the LION database. Point color indicates the enrichment *p* value, and point size represents the number of enriched differential lipids.

Based on lipid class classification, we analyzed and compared the structural differences of lipids between the DME vs. ARC groups (Figure 6A1) and the DME vs. DC groups (Figure 6B1). We screened differential lipids with a correlation coefficient greater than 0.8 and a significant correlation *p* value of less than 0.05 for community classification, and displayed the significant correlations between different lipids through a lipid correlation network diagram (Figures 6A2,B2). In the aforementioned correlation network study, for the top 5 classifications where the number of lipids differed by more than 10, we used the LION lipid ontology database to perform functional enrichment analysis of lipids. Through functional enrichment analysis of these differential lipids, we found that only the DME vs. DC comparison in community 1 showed significant enrichment in the following four categories: sphingolipids, ceramides, endoplasmic reticulum (ER), plasma membrane, and N-acylsphingosines (ceramides) [SP0201] (Figure 6B3). The main components of the lipids enriched in these four categories are Cer (d18:1_16:0); Cer (d18:1_22:0); Cer (d18:1_26:0); and Cer (d35:0).

## 4 Discussion

Previous studies have expanded our understanding of DME by examining various aspects, such as aqueous humor factors (7), proteomics (14), metabolomics (15) and genetics (8). However, an in-depth study or analysis of aqueous humor lipidomics in DME has still not been fully investigated. Consequently, the precise function of lipid metabolism in DME is still not well understood. In this study, we performed LC–MS/MS analysis of aqueous humor from patients with ARC, DC, and DME to determine the lipid profile of their aqueous humor and conducted OPLS-DA and differential lipid analysis. Our main focus in screening is on lipid compounds with *p* < 0.05 and VIP > 1, aiming to identify possible biomarkers for the development and diagnosis of DME. To our knowledge, there have been no previous records of lipidomic research on aqueous humor in DME. This research revealed notable differences between groups in DME vs. DC and DME vs. ARC, and we also found differential lipids that could serve as potential biomarkers.

### 4.1 Sphingolipids

Sphingolipids are crucial structural components of the cell membrane, with their synthesis and degradation balance maintaining membrane stability (16). Sphingolipids, as molecules with potent bioactivity, participate in metabolic regulation and cell signaling, triggering a series of cellular stress responses. The systemic circulation of sphingolipids may act as intermediary indicators for glucose balance imbalances, contributing to the emergence of diabetes and its complications (17).

#### 4.1.1 Ceramide (Cer)

Cer is considered the central node of the sphingolipid metabolism pathway, being a key player in lipid signaling. The fatty acid chain determines the different types of ceramides and their various functions in cells. Research indicates a link between alterations in ceramide levels are associated with apoptosis and cell death. In the process of retinal photoreceptor cell survival and death, ceramide metabolism plays a crucial role. In individuals with insulin resistance, ceramide is intimately associated with inflammatory cytokines (18, 19). However, there may be heterogeneity in the relationship between ceramide subtypes and metabolic profiles. Although serum ceramide subtypes increase in obese populations, the levels of Cer (d18:1_16:0) decrease (20). No prior reports have detected Cer in DME aqueous humor. Conversely, research contrasting the lipid profiles of aqueous humor in diabetic and non-diabetic individuals revealed a decrease in Cer concentration in diabetic aqueous humor (10).

This research indicated a general decline in Cer levels in the aqueous humor between DC vs. ARC, DME vs. ARC, and DME vs. DC all showed an overall downward trend, indicating the vital role of Cer in the development and advancement of DC and DME in diabetics. This study also found that the four lipid compounds Cer (18:1_16:0), Cer (18:1_22:0), Cer (18:1_26:0), and Cer (35:0) are at lower levels in ARC, DC, and DME. Our comparative analysis of lipid function enhancements for DME and DC also revealed a notable increase of the previously mentioned four lipid substances in the ER and cellular membrane. The ER has various metabolic and biosynthetic functions. ER stress can be induced by inflammation and ischemia. According to relevant reports, in the aqueous humor of patients with DME, the ER stress marker glucose-regulated protein 78 (GRP78) shows an upward trend and is associated with VEGF (21). Cer (d18:1_16:0) in plasma is related to geographic atrophy in age-related macular degeneration and fibrosis in diabetic nephropathy (22, 23). Cer (18:1_22:0) is an independent risk factor for DR and it is also associated with high-sensitivity C-reactive protein levels in DM patients (6, 24). Consequently, Cer, stress in the ER, and VEGF might collaboratively contribute to the development of DME in diabetics, yet additional investigation is required.

#### 4.1.2 Sphingosine-1-phosphate (SPHP, S1P)

SPHP, a metabolite of cellular sphingolipids. It controls cell growth, differentiation, survival, and death, making it a potential target for retinal neovascular diseases (25). SPHP transmits signals via G protein-coupled receptors, playing a role in regulating vascular tension and endothelial barrier function, and contributes to the regulation of vascular permeability. Its receptors are high in the retina and subretinal structures (26). For individuals who are suffering from proliferative diabetic retinopathy (PDR), the Müller cells exert control over endothelial cells via exosomes, focusing on the S1P1/AKT/VEGFR2 pathway, activating VEGFR2 phosphorylation and internalization, thereby promoting abnormal vascular growth (27). The biological response of SPHP often differs from Cer, where their balance is crucial for cell survival and apoptosis (28).

Our research revealed that SPHP levels in aqueous humor rose in both DME vs. DC and DME vs. ARC, alongside a general decrease in Cer concentration. In the differential lipid correlation analysis, especially in DME vs. DC, a significant negative correlation between Cer and SPHP was observed, consistent with the previous understanding of these two components. Metabolomics analysis of DME aqueous humor confirmed that SPHP, as a metabolite associated with prolonged oxidative stress and glycation, showed higher level in DME aqueous humor (29), which is consistent with the upregulation of SPHP detected in this study’s lipidomics analysis. The increase in SPHP in DME aqueous humor may be related to the degradation of glycosphingolipids from damaged cells. The specific role of SPHP in DME is still not very clear.

#### 4.1.3 Sphingomyelin (SM)

SM forms part of sphingolipids, acting as both an active precursor for Cer and a buffering entity. The results of *in vitro* experiments indicate that SM and SPHP exacerbate the damage to retinal microvascular endothelial cells in rats caused by high sugar levels and promote the development of DR through oxidative stress pathways (30). Analysis of vitreous lipidomics in type 2 diabetes shows that the total content of SM is elevated (10). In this study, there are differences in the concentration of various structures of SM in the aqueous humor of DME vs. ARC. Specifically, the level of SM (d34:1), SM (d40:1), and SM (d40:2) have decreased. However, the concentration of these three lipid compounds shows an upward trend in the aqueous humor of NVG patients caused by PDR, suggesting that they may be involved in disease formation through different pathways (31). When contrasting DME with DC, a rise in SM and a fall in Cer and ChE levels are observed. Subsequent correlation studies reveal an inverse relationship between SM and Cer.

### 4.2 Phospholipids

#### 4.2.1 Phosphatidylcholine (PC)

PC is the most abundant phospholipid in cells and is associated with metabolic disorders. In Bruch’s membrane, the levels of PC are comparatively high and are associated with the activation of the complement system and inflammatory responses (32). Moreover, oxidized PC in the vitreous can increase the expression level of VEGF in RPE cells (33). PC may act as a mediator between inflammatory bowel disease and DR (34). In this study, significant differences were found in PC content, when we contrast DME with DC patients’ aqueous humor, showing an upward trend in DME. PC (16:0_18:1), PC (16:0_18:2), PC (16:0_20:4), PC (18:0_20:4), and PC (34:2_36:1) show an increasing trend in DME vs. ARC. Notably, these differential lipid compounds also display significant differences and an upward trend when comparing the aqueous humor of NVG patients caused by PDR to the control group (31). The metabolic dysregulation of PC may be involved in the complications of diabetes and the pathogenesis of DME.

#### 4.2.2 Lysophosphatidylcholine (LPC)

LPC is derived from PC in the cycle and plays a role in the development of endothelial dysfunction. LPC (18:1) affects endothelial cells and can induce optic nerve function restoration in mouse models through oligodendrocyte maturation and myelin regeneration, thus providing protective effects on the optic nerve (35, 36). Additionally, plasma LPC (18:1) is a potential biomarker for adolescent obesity (37). VEGF can stimulate LPC (18:1) expression and increase interleukin-8 (IL-8) release, promoting an inflammatory microenvironment and inducing damage. Post anti-VEGF therapy in NVG sufferers, there was a reduction in LPC concentration in the aqueous humor (31). This study found out that LPC (18:1) exhibits significant variability and a downward trend in DME vs. ARC aqueous humor, and also shows significant differences and a downward trend in DC vs. ARC aqueous humor. Therefore, we speculate that LPC (18:1) might be involved as a potential biomarker in the metabolic processes of DC and DME.

### 4.3 Neutral lipids

#### 4.3.1 Cholesterol ester (ChE)

There is a connection between ChE and oxidative stress, which can regulate microglia, macrophages, and others, thus participating in immune and inflammatory responses (38, 39). ChE metabolism in the retina is a highly complex biological process, and once damaged, it may lead to dysfunction in the RPE-Bruch membrane-choroid complex (40). A significant correlation has been observed between the increase in blood ChE levels and DME (41). The rising level of ChE in the aqueous humor of glaucoma, PCV, and Pseudoexfoliation syndrome (PEX) are also observed (42–44). However, in this study, only ChE (20:4) showed a difference in the aqueous humor of DME vs. DC and DME vs. ARC, with both experiencing a decrease. Prior to this, there were no documented instances of ChE (20:4) in aqueous humor; but elevated levels of this lipid were observed in the blood serum of individuals with Ovarian Hyperstimulation Syndrome (OHSS). Additionally, in studies comparing differential lipids in DME vs. DR, ChE showed a significant positive correlation with Cer. ChE (20:4) may be involved in the formation of DME through multiple pathways, but there is currently no clear research on its specific mechanisms.

#### 4.3.2 Triglyceride (TG)

TG is the main form of fatty acid storage and transportation within cells and in plasma (45). There is an association between the triglyceride-glucose (TyG) index and the risk of DR (46). Compared to other lipids, a greater concentration of TG was found in the aqueous humor of patients with DME and patients with DC. This aligns with Jiawei Wang et al.’s previous research, which identified an elevated TG percentage in the aqueous humor of DC patients relative to the control group (10). Patients with high myopia also had relatively higher TG content in their aqueous humor, which aligns with our findings (12). Moreover, although the TG proportion was the same in the aqueous humor samples of both the DME and DC groups in this study, we observed differences in the increase and decrease of various TG types, suggesting a complex regulatory role of TG in the pathogenesis of DME. Previously, no detection of TG in the aqueous humor of DME patients had been reported. In the aqueous humor of NVG patients secondary to PDR, TG (16:0_18:1_18:2) was found to be upregulated compared to the control group (31). The research also noted significant variances in TG (16:0_18:1_18:2) between DME and ARC aqueous humor, indicating an increasing pattern. Chung YR and colleagues, in their research on plasma TG in type 2 diabetes patients, suggested that elevated plasma TG levels might act as an indirect indicator of DME, and statins might prevent the occurrence of DME and the progression of DR (47). Benarous R and others discovered that total cholesterol and triglycerides were independently associated with cystoid macular edema (CME), but unrelated to DR, the severity of DME, or macular thickness (48). Overall, the imbalance in TG metabolism is linked to the formation of DR and DME, yet the precise process behind this is still unclear.

## 5 Conclusion

In our study, we conducted an in-depth analysis of the aqueous humor lipidomics of DME patients for the first time, using statistical and bioinformatics approaches to explore lipid biomarkers that may be associated with the pathogenesis of DME. The lipidomic analysis of DME aqueous humor revealed a number of notable distinct alterations. Metabolic changes in sphingolipids (Cer, SPH, SPHP, SM), phospholipids (LPC, PC), neutral lipids (ChE, DG, TG), and fatty acyls (AEA, PMe, WE) may play a crucial role in the occurrence and development of DME, providing new insights into its pathological mechanisms. The identified differential lipid compounds offer new directions for studying the pathogenesis of DME and lay a solid foundation for further exploration of its complex biochemical mechanisms. These differential lipid compounds may have the potential to serve as biomarkers for the diagnosis, assessment, and treatment of DME, potentially leading to new therapeutic strategies, thereby offering precise guidance for future disease management strategies.

In fact, through reflection and analysis, we have found that our research does indeed have many limitations. First, due to the constraints of surgical procedures and ethical considerations, the number of aqueous humor samples was relatively small. This led to a reduced capability for lipidomics analysis. To verify the reliability of the identified biomarkers, a larger sample size is necessary. During the OPLS-DA analysis on DC and ARC, the validation parameters for the model were less than ideal, suggesting minimal group variability, possibly due to the limited number of samples. Nevertheless, we still identified 27 lipids with significant differential concentration between DC and ARC through the analysis. Secondly, another limitation is that all patients in our study came from a single research center, Nanjing Drum Tower Hospital, which may restrict the generalizability of the results. Despite these limitations, we identified numerous notable lipid variances. For the confirmation and enhancement of our results, subsequent research must incorporate additional samples and clinical trials from various institutions. Third, due to the absence of treatment measures or lipidomics alterations in aqueous humor post-treatment, the exact processes connecting aqueous humor lipid shifts with pre- and post-therapy remain unclear. Future comprehensive exploratory research ought to include forward-looking cohort studies and practical investigations to ascertain if these varied lipids could act as focal points or prognostic markers for DME therapy.

## Data Availability

The original contributions presented in the study are publicly available. This data can be found here: (https://www.ebi.ac.uk/metabolights/reviewer263ce77f-eec7-49de-9e0e-a59ec04bec0b / MTBLS12205).
